# The Natural Compound Dehydrocrenatidine Attenuates Nicotine-Induced Stemness and Epithelial-Mesenchymal Transition in Hepatocellular Carcinoma by Regulating a7nAChR-Jak2 Signaling Pathways

**DOI:** 10.1155/2022/8316335

**Published:** 2022-01-24

**Authors:** Ching-Li Li, Chien-Che Wang, Hsin-Te Pai, Stan-Ley Tu, Pin-Yuan Hou, Chien-Yu Huang, Ming-Te Huang, Yu-Jia Chang

**Affiliations:** ^1^Graduate Institute of Clinical Medicine, College of Medicine, Taipei Medical University, Taipei 110, Taiwan; ^2^Department of Surgery, Pojen General Hospital, Taipei City 105, Taiwan; ^3^Department of Surgery, Taipei Medical University, Shuang Ho Hospital, New Taipei City 235, Taiwan; ^4^Department of Surgery, School of Medicine, College of Medicine, Taipei Medical University, Taipei 110, Taiwan; ^5^Department of Pathology, Wan Fang Hospital, Taipei Medical University, Taipei 116, Taiwan; ^6^Division of Colorectal Surgery, Department of Surgery, Taipei Medical University Shuang Ho Hospital, New Taipei City 235041, Taiwan; ^7^Department of Surgery, Xin Tai General Hospital, New Taipei City 235, Taiwan; ^8^Cancer Research Center and Translational Laboratory, Department of Medical Research, Taipei Medical University Hospital, Taipei Medical University, Taipei 110, Taiwan; ^9^Cell Physiology and Molecular Image Research Center, Wan Fang Hospital, Taipei Medical University, Taipei 116, Taiwan

## Abstract

**Background:**

Exposure to nicotine has been observed associated with tumor progression, metastasis, and therapy resistance of many cancers. Hepatocellular carcinoma (HCC) is one major cancer related to the liver and the most difficult to treat malignancies worldwide. The underlying mechanism of nicotine in the stimulation of HCC tumorigenesis is still not studied well.

**Methods:**

Classically, nicotine binds to nicotinic acetylcholine receptors (nAChRs) and induces many downstream cancer-associated signaling pathways. Big data analysis is used to explore the importance of a7nAChR-Jak2 axis in the progression of hepatocellular carcinoma. Bioinformatic analysis was performed to determine gene associated with a7nAChR-Jak2 axis of HCC patients. Biological importance of a7nAChR-Jak2 axis was investigated in vitro (Hun7 and HepG2 cell lines), and athymic nude mouse models bearing HepG2-HCC cells xenografts were established *in vivo*.

**Result:**

We found that nicotine exposure stimulated the HCC tumorigenicity by inducing the expression of one of the key nAChRs subunit that is *α*7nAChR as well as the expression of Janus kinase (JAK)-2. In both the in vitro and in vivo studies, the reduced overexpression of *α*7nAChR and increased sensitization of HCC towards treatment is observed with dehydrocrenatidine (DHCT), a novel and potent JAK family kinase inhibitor. Interestingly, DHCT treatment results in the reduction of the epithelial-mesenchymal transition process which leads to a significant reduction of clonogenicity, migratory, and invasive ability of HCC cells. Moreover, DHCT treatment also inhibits the cancer stem cell phenotype by inhibiting the tumor-sphere formation and reducing the number of ALDH1+ cells population in nicotine-stimulated HCC cells.

**Conclusions:**

Taken together, the presented results indicate the positive effect of inhibition of nicotine induced overexpression of *α*7nAChR and JAK2, unique to HCC. Thus, these findings suggest the nicotine effect on HCC progression via *α*7nAChR-mediated JAK2 signaling pathways, and DHCT treatment enhances the therapeutic potential of HCC patients via overcoming/reversing the effect of nicotine in HCC patients.

## 1. Introduction

Currently, over a billion people in the world, including approximately 19% of adults, actively smoke tobacco [1]. Smoking releases more than 4000 harmful chemicals, which adversely affects almost all organs [2], including the liver [2]. Smoking is a well-known risk factor for many diseases, including cancers [3]. However, how cigarette smoking contributes to the development of liver cancer remains underinvestigated.

Nicotine, which is a major addictive chemical component of cigarette smoking, poses much serious health; this compound influences numerous biological function such as modulation of gene expression, DNA damage, oxidative stress, proliferation, apoptosis, angiogenesis, and regulation of secretion of hormones [4]. This compound exerts its effects via stimulation of the nicotinic acetylcholine receptors (nAChRs) that are ligand-activated ion channels [5]. Among various subtypes of nAChR receptors, alpha7-subtype of nAChR (*α*7nAChR) has been seen to exert special significance on the cellular process [6, 7]. *α*7nAChR is expressed and it is involved in regulation of a variety of human cancer and normal cells and tissues, such as liver cancer [6, 7]. It modulates many cancer-associated properties in most cancers and interestingly plays a vital role in the regulation of inflammatory markers expression, which regulates various other pathological conditions [8–11]. Such a broad role of *α*7nAChR and the effect of nicotine have a significant impact in defining the long-term faith of different cancers.

It is noticed that the number of different nicotine-related malignancies is on the rise [12]. Among, them, hepatocellular carcinoma (HCC) is one of the most common, and often diagnosed malignancies, which ranks number the second leading reason of cancer-related mortality worldwide with approximately 800,000 new cases reported every year [13]. Despite advancements in early detection and diagnostic techniques and treatment modalities, the prognosis of HCC patients remained poor even after the curative liver resection [14]. Several epidemiologic studies have reported that smoking tobacco is connected with HCC development [15]. However, the biological effect of tobacco and the underlying molecular mechanism in HCC remain elusive.

In the previous study by our group, we demonstrated by applying the HCC patient's clinical samples that smoking is an independent risk factor for HCC progression through the *α*7nAChR and JAK2 signalling [16]. In the cholinergic system, *α*7nAChRs facilitates many different pathways, it is extensively studied in association with the anti-inflammatory pathways [17]. In monocytes and macrophages, *α*7nAChR triggers and attracts the cytosolic JAK2 protein, leading to the initiation of the formation of a heterodimer complex that triggers STAT3 signal transduction [18]. The JAK2/STAT3 signaling pathways also play a critical role in the growth, proliferation, and metastasis of cancers [19]. The JAK2-modulated STAT3 transcription factors are involved in many processes, such as immune response, inflammation, and cancer progression, by activating proinflammatory cytokines, growth factors, and oncogenes [20, 21]. Apart from *α*7nAChR negative regulatory role in the inflammatory response, the role of *α*7nAChR together with JAK2, in connection with exposure to nicotine in HCC, remains unclear. This prompted us to identify new potential drugs to overcome the effects of nicotine on HCC. For example, the natural compound dehydrocrenatidine (DHCT), a novel and potent JAK family kinase inhibitor that modulates JAK/STAT3 expression [22] in HCC, can sensitize HCC cells against nicotine.

In the current study, we investigated the functional significance of nicotine and *α*7nAChR in HCC progression and therapy resistance, the molecular mechanisms underlying its oncogenic role in HCC, and the potential of DHCT treatment to reverse the effects of nicotine in HCC.

## 2. Materials and Methods

### 2.1. Chemicals and Reagents

Dehydrocrenatidine, ≥98% purity, was purchased from AOBIOUS (CAS No: 65236-62-6, AOBIOUS), dissolved in dimethyl sulfoxide (DMSO) to 20 mg/mL as stock solution and kept at -20°C until use. Nicotine, ≥99% (GC), liquid, purity was purchased from Sigma-Aldrich (CAS No: 54-11-5).

### 2.2. Cell Lines and Culture Media

The human Huh7 and HepG2 HCC cell lines were purchased from the American Tissue Culture Collection and cultured in Roswell Park Memorial Institute (RPMI) 1640 Medium (Thermo Fisher Scientific, Waltham, MA, USA) supplemented with 10% fetal bovine serum (FBS) and 1% penicillin-streptomycin (Invitrogen, Life Technologies) at 37°C in a 5% humidified CO_2_ incubator.

### 2.3. Microarray and RNAseq Preprocessing and Analysis

Gene expression profiles, GSE14323, were downloaded from the Gene Expression Omnibus database (http://www.ncbi.nlm.nih.gov/geo/), and expression of *α*7nAChR and JAK2 was analyzed using the Oncomine platform (https://www.oncomine.org/resource/) [23].

### 2.4. Cell Viability Test

The DHCT stock was prepared by dissolving 20 mg/mL of the mixture in DMSO. The stocks of each drug were stored at −20°C until use. The effects of DHCT on cell proliferation were detected using the Cell Counting Kit-8 (CCK-8) assay (Dojindo Laboratories, Kumamoto, Japan), according to manufacturer's instructions.

### 2.5. Cell Cycle Analysis

The distribution of cell cycles was determined by staining cells with the propidium iodide (PI), after treatment, according to manufacturer's instructions. To determine the effect on the cell cycle, HCC cells were exposed to DHCT for 48 h. Next, the cells were washed, fixed with 70% ethanol, washed again, resuspended, and stained with 10 *μ*g/mL of PI in PBS for 30 min at room temperature in the dark. The cells were analyzed through by flow cytometry (Becton Dickinson, Mountain View, CA, USA), and the population of cells in each phase was counted.

### 2.6. Aldehyde Dehydrogenase Assay

Aldehyde dehydrogenase (ALDH) activity in HCC cells was assayed using the ALDEFLUOR kit according to manufacturer's instructions (STEMCELL Technologies, Durham, NC, USA). The cells that showed positive activity for ALDH1 were isolated and analyzed. Briefly, the human HCC cell lines Huh7 and HepG2 were suspended at 1 × 106 cells/mL in ALDEFLUOR assay buffer containing the ALDH substrate (BAAA, 1 *μ*mol/L per 1 × 106 cells) and incubated for 40 min at 37°C. Cells incubated with the ALDEFLUOR substrate and treated with 50 mmol/L of diethylaminobenzaldehyde (DEAB), a specific ALDH inhibitor, were used as a reference control. Cells stained with PI alone were used as negative controls, and ALDEFLUOR-stained cells treated with DEAB and those stained with the secondary antibody alone were considered viable.

### 2.7. Western Blotting and Quantitative Reverse-Transcription Polymerase Chain Reaction

After the corresponding treatment, the HCC (Huh7 and HepG2) cells were washed with PBS and then lysed in RIPA lysis buffer. Cellular protein lysates were isolated using a protein extraction kit (QIAGEN, USA) and quantified using the Bradford protein assay kit (Beyotime, Beijing, China). Approximately 20 *μ*g of sample from different experiments was loaded and subjected to SDS-PAGE by using the Mini-PROTEAN III system (Bio-Rad, Taiwan). Separated proteins were transferred onto a polyvinylidene fluoride (PVDF) membrane by using the Trans-Blot Turbo Transfer System (Bio-Rad), which was followed by blocking with Tris-buffered saline plus skimmed milk. These PVDF membranes were then probed with the respective primary antibodies followed by the secondary antibody. An enhanced chemiluminescence detection kit was used to detect the proteins of interest. Images were captured and analyzed using the UVP BioDoc-It system (Upland, CA, USA). Quantitative reverse-transcription (RT) polymerase chain reaction (PCR) was performed by isolating total RNA using TRIzol-based protocol (Life Technologies) provided by the manufacturer. In brief, 1 *μ*g of total RNA was reverse transcribed using a QIAGEN OneStep RT-PCR kit (QIAGEN, Taiwan), and PCR was performed using a Rotor-Gene SYBR Green PCR kit (400; QIAGEN, Taiwan). The Commercial antibodies and primers used in this study are shown in Supplementary Table S1 and Supplementary Table S2, respectively.

### 2.8. Colony Formation Assay

As per the protocol for colony formation assay described previously [24], 400 liver cancer cells were seeded in six-well plates and treated with DHCT. The cells were allowed to grow for another week and then harvested, fixed, and counted. The image was captured and quantified using ImageJ software (https://imagej.nih.gov/ij/download.html).

### 2.9. Wound Healing Migration Assay

The cells were seeded in six-well plates (Corning, Corning, NY, USA) with RPMI 1640 medium containing 10% FBS after the cells reached 95%–100% confluence. A scratch along the median axis was then made with a sterile yellow pipette tip across cells. Cell migration pictures of cells with or without DHCT treatment were captured at 0 and 48 h under a microscope and analyzed using NIH Image J software (https://imagej.nih.gov/ij/download.html).

### 2.10. Matrigel Invasion Assay

Cells (3 × 10^5^) were seeded in 24-Transwell chambers with an 8 *μ*m pore membrane coated with Matrigel in the upper chamber of the Transwell system containing serum-free RPMI 1640 medium. The lower chamber of the Transwell system contained the medium with 20% FBS. After incubation at 37°C for 6 h, noninvaded HCC cells on the upper side of the membrane were carefully removed with a cotton swab, and the invaded cells were stained with crystal violet dye, air-dried, and photographed under a microscope. Images were analyzed using NIH Image J software (https://imagej.nih.gov/ij/download.html).

### 2.11. Sphere Formation Assay

Cells (5 × 10^3^/well) were plated in ultralow-attachment six-well plates (corning) containing stem cell medium consisting of serum-free RPMI 1640 medium supplemented with 10 ng/mL of human basic fibroblast growth factor (bFGF; Invitrogen, Grand Island, NY, USA), 1× B27 supplement, and 20 ng/mL epidermal growth factor (Invitrogen). The medium was changed every 72 h. After 14 days of incubation, the formed spheres were counted and photographed.

### 2.12. Animal Studies

This study was approved by the Institutional Animal Care and Use Committee (IACUC) of Taipei Medical University. All procedures were performed according to guidelines of IACUC, and all efforts were made to minimize animal suffering and the number of animals used. Twenty-seven male athymic nude mice at 4 weeks of age were used for this study. The mice were divided into three groups (*N* = 6 per group) and repeat experiments 3 times. The mice were maintained under pathogen-free conditions and were provided with sterilized food and water. The antiproliferative effect of DHCT on HepG2-HCC cells in vivo was investigated by using HepG2 cell xenograft mouse model of human HCC carcinoma. First, 1 × 10^6^ HepG_2_ cells along with proper controls were subcutaneously injected into the right flank near the hind leg of each nude mouse. When the tumor was palpable (tumor volume of approximately 100 mm^3^), they were randomly divided into control (100 *μ*L of normal saline [NS] by intraperitoneal injection plus 100 *μ*L of 1% DMSO), nicotine was administered, and the role of DHCT treatment in overcoming the effect of nicotine (200 mg/kg/day by intraperitoneal injection plus 100 *μ*L of 1% DMSO and 0.5% CMC-Na sterile water). The treatments were performed 5 times/week for 4 weeks. Tumor volume was measured using a standard vernier calliper, the tumor volume was calculated using the established formula. Tumor volume = 1/2 (length × width2) in mm^3^ [25]. Upon the completion of the experiments, all the mice were euthanized by CO_2_ asphyxiation followed by cervical dislocation, and the tumors were then immediately removed, weighted ex vivo and photographed for further analysis.

### 2.13. Immunohistochemistry (IHC) Staining

The tumor tissue was fixed in 10% neutral-buffered formalin, embedded in paraffin wax, and cut into sections of 3 *μ*m thickness. Then, the sections were immunohistochemically stained with anti-Ki67 and anti-cleaved (cl)-Caspase-3 antibodies (Cell Signaling Technology) at 4°C overnight. Slides were rinsed with phosphate-buffered saline (PBS) and incubated at room temperature for 30 minutes with biotinylated goat anti-mouse IgG secondary antibody (Dako, Glostrup, Denmark). After washing in Tris-hydrochloride acid buffers (TBS), the slides were incubated with streptavidin-peroxidase reagent (Dako) and treated with 3,3′-diaminobenzidine (DAB; Sigma Aldrich; Merck KGaA) for 5 minutes. Finally, sections were rinsed with ddH2O and counterstained with hematoxylin. Slides were observed under microscope, with the selection of 5 fields of view randomly. The final percentage of positive cells was calculated with the Motic Image software (version 1.2; Micro-Optical Group Co.)

### 2.14. Terminal Deoxynucleotidyl Transferase dUTP Nick-End Labeling (TUNEL) Assay

Apoptotic cell death in the HCC tumor tissue was determined by TUNEL assay using an Apoptag® Peroxidase In Situ Apoptotic Detection Kit (Roche according to manufacturer's protocol). Quantification was performed by calculating the percentage of TUNEL-positive cells by using a fluorescence microscope. The results are expressed as the mean number of TUNEL-positive apoptotic HCC cells in each group.

### 2.15. Statistical Analysis

All assays were performed at least three times in triplicate. Values are expressed as mean ± standard deviation (SD). Comparisons between groups were estimated using Student's *t*-test for cell line experiments or the Mann–Whitney *U*-test for clinical data. The Kaplan–Meier method was used for survival analysis, and the difference between survival curves was tested with a log-rank test. All statistical analyses were performed using IBM SPSS Statistics for Windows, version 20 (IBM, Armonk, NY, USA). *p* < 0.05 was considered statistically significant.

## 3. Results

### 3.1. Nicotine Augments Cell Proliferation Abilities of Huh7 and HepG2 Human HCC Cells through *α*7nAChR-JAK2

**Figure 1:**
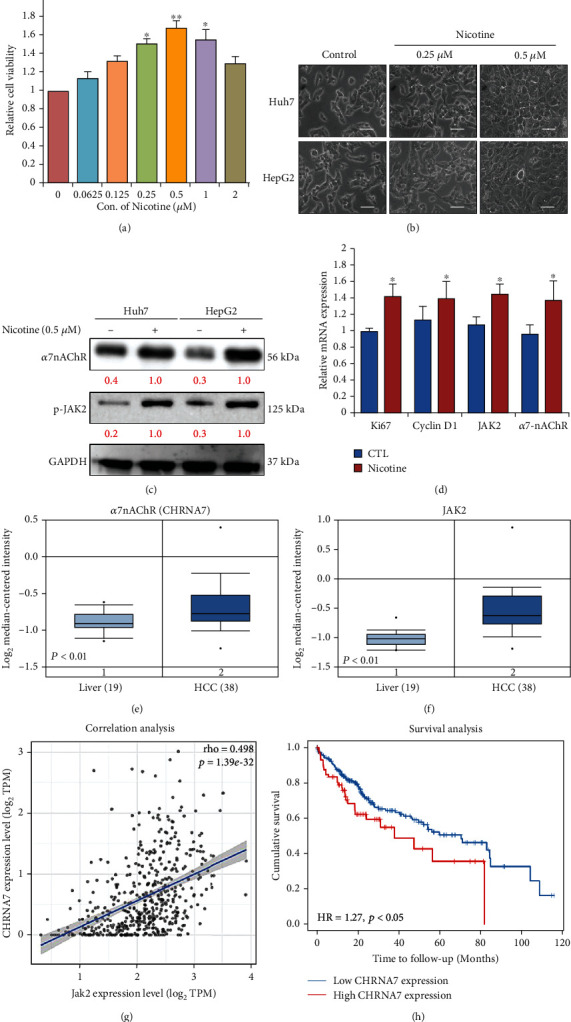
Nicotine treatment promotes cell proliferation of HCC cells. (a) Huh7 and HepG2 cells (5 × 10^4^ cells) were treated with the indicated concentrations of nicotine for 48 h. The cell viability of Huh7 and HepG2 was determined by the CCK-8 assay. (b) Optical microscopy showed nicotine treatment at 0.25-0.5 *μ*M dose-dependently induced morphological changes of HCC (Huh7 and HepG2) cells. Scale bar 100 *μ*m. (c) Huh7 and HepG2 cells stimulated with 0.5 *μ*M nicotine for 48 h were harvested, and the effects of nicotine on *α*7nAChR and JAK2 expression were determined by western blot analysis. (d) Real-time PCR analysis of Ki67, Cyclin D1, JAK2, and *α*7nAChR mRNA in HCC cells. (e, f) Expression of *α*7nAChR (CHRNA7) and JAK2 in liver cancer (GSE14323). (g) Correlation analysis of *α*7nAChR (CHRNA7) expression with the JAK2 expression in LIHC TCGA patient data. (h) Kaplan-Meier analysis of expression of *α*7nAChR (CHRNA7) and JAK2 for LIHC patients based on the result from the public TCGA data. GAPDH was used as an internal control. Bar represent the mean ± SD values of three independent experiments; ^∗^*p* < 0.05; ^∗∗^*p* < 0.01.

First, we examined the effect of nicotine on HCC cell proliferation, both the Huh7 and HepG2 cells were titrated with the 0.0625 to 2 *μ*M concentration of nicotine for 48 h, and cell viability of these cells was determined by CCK-8 assay. The result showed that, as compared to control (PBS treated), nicotine treatment augments the human HCC (Huh7 and HepG2) cell proliferation (Figures 1(a) and 1(b)). *In vitro*, nicotine stimulates the proliferation of HCC cells through *α*7nAChR. Meanwhile, Western blot results also indicated, as shown in Figure 1(c), nicotine treatment at 0.5 *μ*M and stimulated the expression of p-JAK2 and *α*7nAChR in both the Huh7 and HepG2 cells. Real-Time Quantitative Reverse Transcription (qRT)-PCR analysis of the mRNA expression level showed the level of Ki67, Cyclin D1, JAK2, and *α*7nAChR in Huh7 and HepG2 cells was significantly higher in the nicotine-treated cells compared to the control-treated group (Figure 1(d); ^∗^*p* < 0.01). Furthermore, bioinformatics analysis of publicly available dataset (GSE14323) by using Oncomine an online tool (https://www.oncomine.org/resource/) [23] also demonstrated (Figures 1(e) and 1(f)) the expression of JAK2 and *α*7nAChR, considerably higher in the HCC patients samples compared to the normal counterpart, whereas the expression of *α*7nAChR correlates positively with the expression of JAK2 expression in HCC patients' samples (Figure 1(g)), and the poor overall survival of HCC patients was observed in patients exhibiting higher expression of *α*7nAChR (Figure 1(h)). All of these in vitro and in silico results indicated the nicotine treatment induces the HCC tumorigenesis by inducing the expression of *α*7nAChR-JAK2.

### 3.2. Nicotine Enhances Migratory, Invasive, Self-Renewal, and EMT Processes of HCC Cells

**Figure 2:**
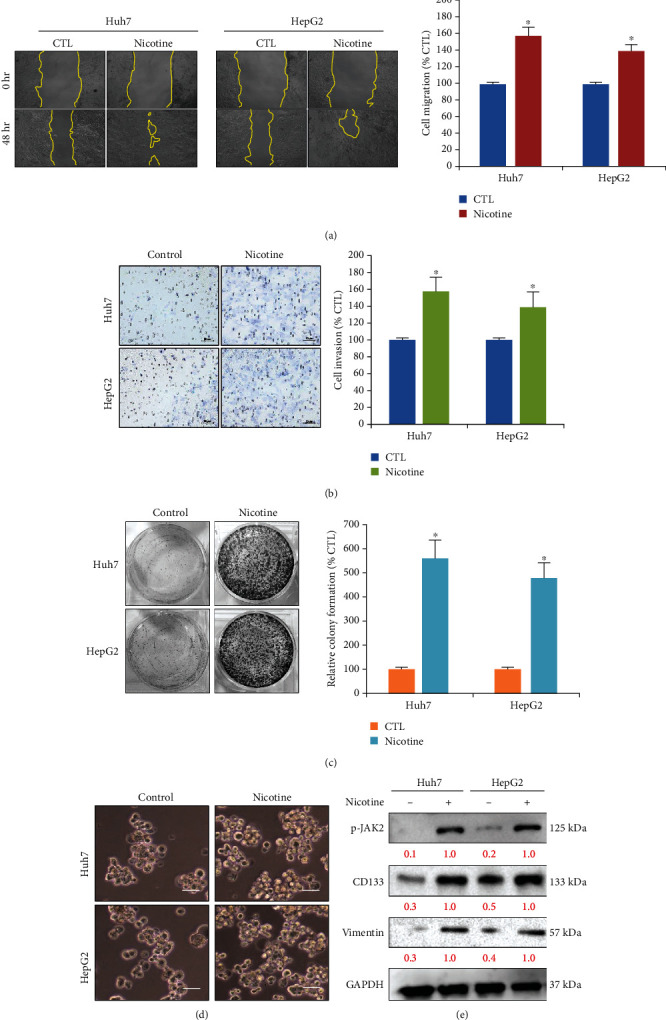
Nicotine induces Huh7 and HepG2 HCC cell migratory, invasive, self-renewal, and EMT properties in vitro. (a, b) Wound healing and Transwell assay, respectively, measured cell migration and invasion, at the concentration of 0.5 *μ*M nicotine for 24 h stimulate both the HCC (Huh7 and HepG2) cells; bar plot indicates the percentage speed of wound healing and invasion between the nicotine and control treatment group. Scale bar 50 *μ*m. (c, d) Nicotine induces self-renewal, i.e., colony-forming and tumor-sphere properties of HCC cells. Scale bar 100 *μ*m. (e) Protein expression by Western blotting in HCC cells treated with nicotine. Nicotine treatment (0.5 *μ*M) efficiently induced the expression of p-JAK2, EMT marker (induced vimentin), and self-renewal (CD133) markers. GAPDH served as the loading control. The bar represents the mean ± SD values of three independent experiments; ^∗^*p* < 0.05 versus controls (Student's *t*-test).

Furthermore, we evaluated the effect to evaluate the effect of nicotine treatment on HCC cell migratory, invasive, self-renewal, metastasis, and other malignant biological behaviour. The result indicates nicotine (0.5 *μ*M)-treated cells after 24 h stimulated the Huh7 and HepG2 cells to migrate more quickly and close the scratch wounds (Figure 2(a)). Furthermore, we conducted a Transwell invasion assay to evaluate the effect of nicotine on the invasive properties of HCC cells. The results demonstrated that both the HCC, Huh7, and HepG2 cells significantly enhanced the ability to invade through the Matrigel matrix, and the number of cells that invaded through the Matrigel matrix was significantly higher after the nicotine treatment, compared to the control (Figure 2(b)). Studies have shown that cancer stem cells (CSCs) are characterized by unlimited proliferative properties and showing uncontrolled cellular growth [23]. Here, we performed the colony-formation and tumor-sphere abilities of nicotine-treated HCC cell (Huh7 and HepG2) results and demonstrated that nicotine treatment significantly induced the colony and tumor-sphere generating abilities of treated HCC cells compared to the control group (Figures 2(c) and 2(d)). The epithelial to mesenchymal transition (EMT) process indicates the tumor invasiveness and angiogenesis of cancer cells. Hence, we investigated the expression of markers associated with the EMT process together with the expression status of p-JAK2 and cancer stem cell markers' (CSCs) markers. Western blot image depicted in Figure 2(e) results demonstrated that the expression level of epithelial-marker vimentin (Vim) and CSC's marker (CD133) was induced together with the expression of p-JAK2 after the nicotine exposure of both the HCC (Huh7 and HepG2) cells.

### 3.3. Dehydrocrenatidine (DHCT) Treatment Reversed the Nicotine Effect on Human HCC Cells via Targeting the Expression of *α*7nAChR-JAK2

**Figure 3:**
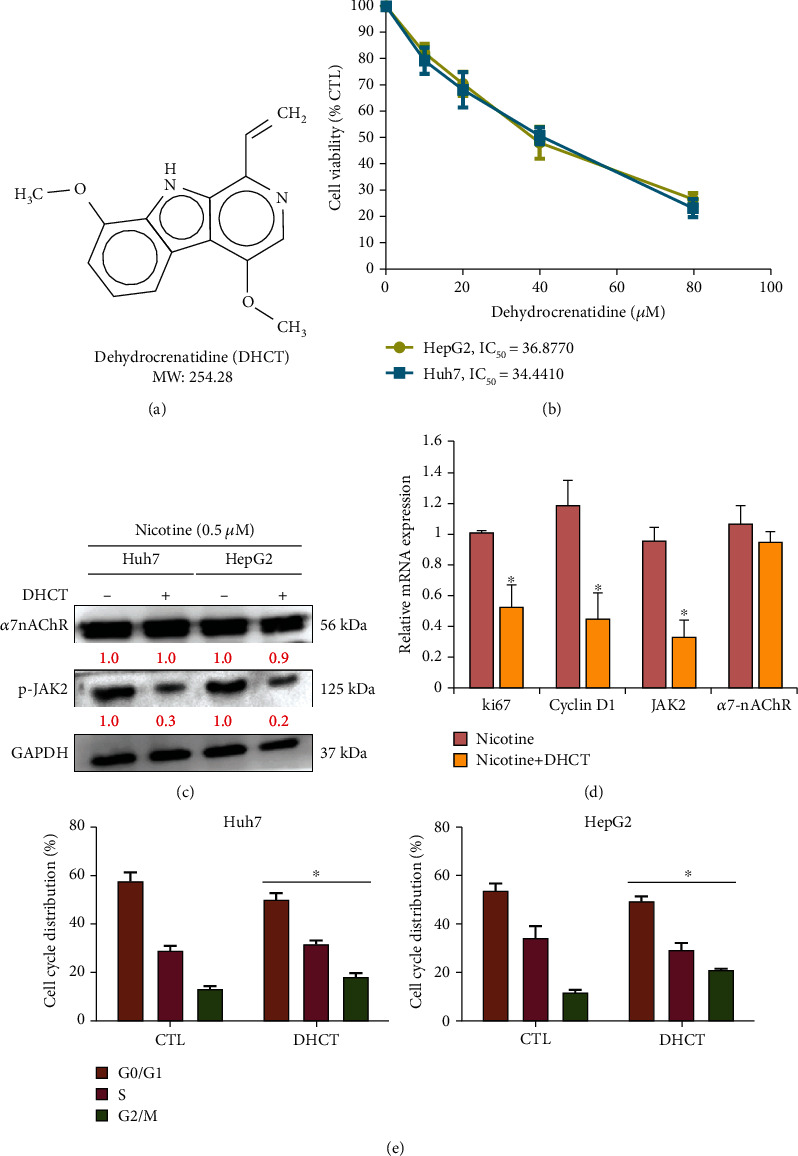
Dehydrocrenatidine (DHCT) modulates the nicotine treatment effect on HCC cells. (a) The image represents the chemical structure of DHCT. (b) Cell viability assay for IC_50_ evaluation of DHCT treatment on both Huh7 and HepG2 HCC cells were determined by CCK-8 assay. (c) The relative reduction in protein expression of *α*7nAChR and p-JAK2 by Western blotting, (d) qRT-PCR analysis denoted the relative reduction at mRNA level of Ki67, Cyclin D1, JAK2, and *α*7nAChR after DHCT treatment on nicotine stimulated cells. (e) DHCT-treated HCC cells (Huh7 and HepG2) cells were harvested; cell cycle was analyzed as the percentage of cells at each stage of the cell cycle after DNA staining with PI. Data from one of the representative experiments (from at least three independent assays); ^∗∗^*p* < 0.01 versus controls (Student's *t*-test).

To examine the potential role of DHCT as a potential drug that targets *α*7nAChR-JAK2 may reverse the effect of nicotine from tobacco smoke in HCC (Huh7 and HepG2) cells. DHCT, a novel and potent JAK family kinase inhibitor, inhibits JAK/STAT3 expression [26] (Chemical structure of DHCT, Figure 3(a)). Furthermore, we treated Huh7 and HepG2 cells with the serial dose of DHCT. As shown in Figure 3(b), the IC_50_ value of DHCT treatment on Huh7 (IC_50_: 34.4 *μ*M) and HepG2 (IC_50_: 36.87 *μ*M) cells sensitizes towards nicotine, to confirm the effect of DHCT effect on the expression of *α*7nAChR and JAK2. The result of Western blot analysis (Figure 3(c)) demonstrated *α*7nAChR and JAK2 expression was significantly inhibited in DHCT treated cells compared to the control (nicotine only treated cells). Interestingly, the mRNA expression levels of Ki67, Cyclin D1, JAK2, and *α*7nAChR significantly inhibited on both the Huh7 and HepG2 HCC-DHCT-treated cells as compared to control-treated (nicotine only) (Figure 3(d); ^∗∗^*p* < 0.01). Furthermore, the cell cycle was analyzed as the percentage of cells at each stage of the cell cycle arrest after DNA staining with PI. The effect of DHCT (IC_20_) on the cell cycle distribution was observed by FACS analysis, as described in Figure 3(e), after 48 h of DHCT exposure. The HCC cells showed a higher percentage of arrest at G0/G1 phase (Huh7: 47%; HepG2: 44%) and a lower number of cells at the G2/M phase (Huh7: 17%; HepG2: 14%) compared to the control nicotine only treated cells, the lower number of cells at G0/G1 phase (Huh7: 51%; HepG2: 49%), and the higher number of cells G2/M phase (Huh7: 10%; HepG2: 8%), indicating the DHCT important role in neutralizing the nicotine effect on HCC cells.

### 3.4. DHCT Treatment Inverses the Nicotine Effect on HCC Cell Migratory, Invasive, and EMT Properties

**Figure 4:**
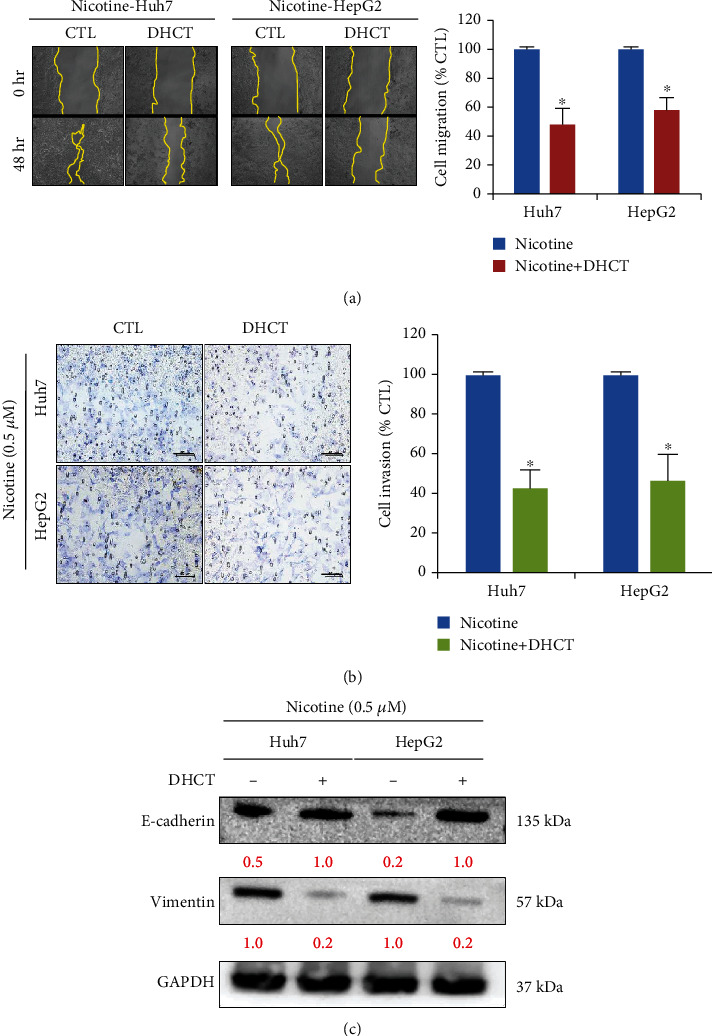
DHCT reversed nicotine induces cell migratory, invasive, and EMT properties in Huh7 and HepG2 HCC cells. (a, b) Wound healing and Transwell assay, respectively, measured cell migration and invasion, after the DHCT treatment on nicotine (0.5 *μ*M) stimulated HCC (Huh7 and HepG2) cells, bar plot adjacent indicates the percentage speed of wound healing and invasion between the DHCT + nicotine and nicotine treatment group. Scale bar 50 *μ*m. (c) Western blot analysis of protein expression on HCC cells treated with DHCT efficiently reduced the expression of EMT process; induced E-cadherin, and reduced vimentin expression observed. GAPDH served as the loading control. Data from one of a representative experiment, (from at least three independent assays); ^∗^*p* < 0.05 versus controls (Student's *t*-test).

Additionally, we examined the effect of DHCT on oncogenic properties of nicotine-stimulated HCC cells. The effect of DHCT on the migratory and invasive properties of HCC cells (Huh7 and HepG2) was investigated. Treatment with DHCT (IC_20_) for 48 h strongly reduced the migration (Figure 4(a)) and invasion (Figure 4(b)) capacity of cells, showing that JAK/STAT3 inhibitor effectively reduced the motility and invasiveness of nicotine stimulated HCC cells when compared with the nontreated control counterpart. EMT plays a key role in the invasion and metastasis of HCC cells [27]. Furthermore, the effect of DHCT on EMT properties and expression of EMT markers, E-cadherin and vimentin, were determined through Western blotting (Figure 4(c)). The results indicated the increased expression of E-cadherin and reduced expression of vimentin. These results advocated the DHCT effects in preventing and neutralizing the effect of nicotine on the migratory and invasive potential of HCC cells.

### 3.5. DHCT Treatment Modulates the Nicotine Effect on Tumorigenicity and Self-Renewal Abilities of HCC Cells

**Figure 5:**
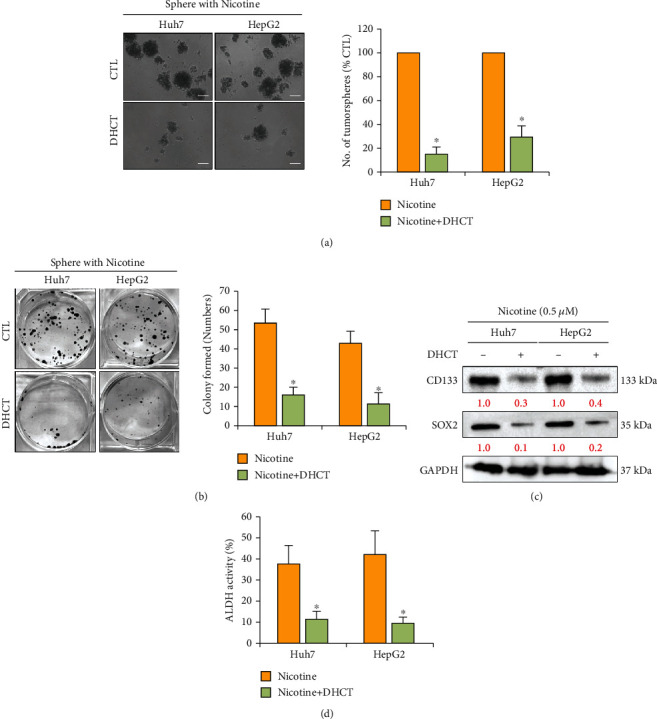
DHCT reversed nicotine effects on tumor sphere and colony-forming abilities of Huh7 and HepG2 HCC cells. (a, b) Representative image of DHCT treatment on the self-renewal, i.e., tumor-sphere and colony-forming properties of HCC cells. Scale bar 100 *μ*m. (c) Detection of the expression of self-renewal (CD133 and SOX2) markers. GAPDH served as the loading control. (d) Representative bar graph of fluorescence-activated cell sorting (FACS) showing reduction of ALDH^+^ cancer stem-like cell population of DHCT-treated HCC cell line compared to nontreated control; ^∗^*p* < 0.05; ^∗∗^*p* < 0.051 versus controls (Student's *t*-test).

To further examine the effect of DHCT in modulating the effect of nicotine on tumorigenic properties of HCC cells, we assayed the colony and tumor sphere formation of the human HCC cell lines Huh7 and HepG2. Colony and tumor sphere formation assays are important for the identification of stemness [28, 29]. DHCT treatment suppressed tumor sphere and colony formation of HCC cells (Figures 5(a) and 5(b)). Interestingly, tumor sphere and self-renewal markers, such as CD133 and SOX2, were significantly reduced at the protein level (Figure 5(c)) after treatment with DHCT compared to nicotine only treated group cells. FACS analysis also demonstrated the reduction in the generation of ALDH1+ cells in DHCT treated cells compared to the nicotine only treated group (Figure 5(d)). These results indicate DHCT potential in inhibiting the self-renewal phenotype of nicotine-stimulated HCC cells.

### 3.6. In Vivo Suppressive Effect of DHCT on Nicotine-Induced HepG2 Tumorigenesis

**Figure 6:**
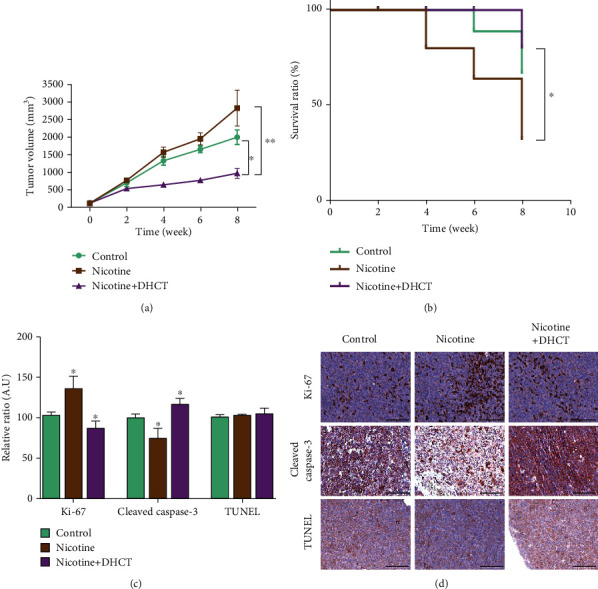
The combination of DHCT+nicotine inhibits tumor growth in HCC mice models. (a) The tumor volume over the time curve was seen in combination treatment, as compared to the nicotine and saline control individual treatment groups. (b) Kaplan–Meier survival curve showed the best overall survival ratio in DHCT+nicotine combination, compared to nicotine only and saline control treatment groups. (c) Comparative real-time PCR analyses showed a significantly reduced Ki-67 expression and induced apoptosis (cl-Caspase-3 and TUNEL-positive cells) mRNA level in combination groups as compared with nicotine and saline counterparts. (d) Representative hematoxylin and eosin (H&E) stained images are shown, and IHC detected the expression of Ki-67 and cl-Caspase-3, and TUNEL apoptotic cells in the tumors from mice were detected. ^∗^*P* < 0.05, and ^∗∗^*P* < 0.01.

After establishing the DHCTs' anti-HCC role on *in vivo* nude mice model. *In vivo* study, we evaluated the DHCT effects using a xenograft mice HepG2 tumor model. After the tumors were palpable in mice, the mice were randomly divided into three groups, control (*n* = 5; saline treatment only), nicotine (*n* = 5; nicotine-treated), and DHCT+nicotine (*n* = 5; DHCT+nicotine-treated) groups. The tumor volume was monitored once per week for another 5 weeks. As depicted in Figure 6(a), the nicotine-treated group mice are showing enhanced tumor growth as compared with that of the saline only control and DHCT+nicotine treatment group, suggesting the combination treatment of DHCT+nicotine leads to a significant reduction in tumor volume (^∗∗^*p* < 0.01). Moreover, the overall survival time was higher in mice treated with the DHCT+nicotine combination compared with that in mice treated with nicotine and nontreated control groups (Figure 6(b)). Moreover, comparative real-time PCR analyses showed (Figure 6(c)) a reduced level of tumor aggressiveness Ki-67 expression markers, induced apoptotic Caspase-3 and TUNEL-positive marker in the DHCT+nicotine combination group in comparison with saline and nicotine treatment. Furthermore, IHC analysis results of tissue sections in mice were consistent with the results of qRT-PCR. IHC result demonstrated that combination treatment of DHCT+nicotine suppresses the Ki-67 marker, i.e., tumor proliferation, oncogenicity, and tumorigenesis, and induced apoptosis was observed as compared with saline and nicotine treatment (Figure 6(d)) animal group.

## 4. Discussion

Nicotine, a major by-product of cigarette smoking, may be responsible for the initiation, progression, and therapy outcomes of various cancers [26]. It influences various biological processes by altering gene expression and inducing oxidative stress, DNA damage, apoptosis, cell proliferation, and angiogenesis [26, 27]. It also contributes to carcinogenesis in various cancer types, affects cancer progression, and is associated with poor prognosis [28, 29]. Nicotine binds to nAChRs and induces many downstream cancer-associated signaling pathways [29]. Epidemiological studies have reported that smoking tobacco significantly increases the initiation and progression of HCC [30].

Nicotine regulates many cancer-associated properties in most cancers and plays a vital role in inflammatory marker expression, which then regulates various other pathological conditions [8–11]. Many malignancies are associated with increased exposure to nicotine [29], notably HCC, which is a common type of liver cancer and is one of the most difficult to treat malignancies worldwide [31].

Our previous study highlighted smoking as an independent risk factor for HCC progression through *α*7nAChR and JAK2 signaling. [16]. In this study, we investigated the mechanisms underlying nicotine's effects on HCC progression through *α*7nAChR and JAK2 signaling and the reversal of those effects by DHCT, a novel kinase inhibitor.

In the present study, we demonstrated that nicotine treatment induced the HCC cells' proliferation, invasion, and self-renewal abilities (Figures 1 and 2) by stimulating and inducing *α*7nACh, JAK2, Ki67, and cyclin D1 expression (Figure 1). Ki67 and cyclin D1 expression indicate the proliferative abilities of cancer cells [32]. Moreover, nicotine exposure significantly modulates the EMT [33], which is associated with invasion, metastasis, and self-renewal (CSCs) [34] of HCC cancer cells (Figure 2). This agrees with recent studies reporting that nicotine exposure stimulates *α*7nAChR expression in cancer cells [35]. de Jonge et al. [18] and Krafft et al. [36] have demonstrated that the expressions of *α*7nAChR and JAK2 are strongly correlated. Notably, *α*7nAChR and JAK2 expression was associated with poor overall survival in patients with HCC [16, 37]. Thus, the *α*7nAChR gene might play a key role in HCC through JAK2 signaling. Furthermore, DHCT significantly inhibited the *in vitro* growth and proliferation of HCC cells and sensitized them to nicotine treatment. DHCT treatment reduced the expression of *α*7nAChR, JAK2, and markers of proliferation and cell cycle arrest (Figure 3). Regarding *in vitro* mobility, migration and invasion are associated with the metastatic potential of HCC cells [38]. Our data confirmed (Figure 4) that DHCT effectively inhibited the effects of *α*7nAChR and JAK2 in nicotine-treated HCC cells, which subsequently reduced their migration/invasion abilities by disturbing the expression of EMT biomarkers (E-cadherin and vimentin) and a transcription factor (Slug).

Often, patients with HCC have chemoresistance, which is enhanced by nicotine exposure (Figure 2). The expression of CSCs is connected to drug resistance [34], which is a major challenge in HCC therapy [39]. CSCs contribute to tumorigenicity, self-renewal abilities, and chemoresistance [40, 41]. Notably, DHCT treatment of HCC cells resulted in the reduced expression of *α*7nAChR, JAK2, and cancer stemness markers (CD133, KLF4, and SOX2) as well as decreased ALDH1 activity (Figure 5), thereby suppressing the colony-forming and tumor sphere generation abilities of HCC cells.

To further confirm the effect of DHCT in modulating and sensitizing the nicotine effect *in vivo*. HepG2 cells were subcutaneously injected into the xenograft mice models. Nicotine alone or in combination with DHCT+nicotine and saline control was administered. Reduction in tumor burden, reduced expression of Ki-67, and induced tumor apoptosis were observed in the DHCT+nicotine treatment group, which results in the reduction/neutralizing effect of nicotine on tumor-initiating abilities in xenograft models (Figure 6).

## 5. Conclusion

**Figure 7:**
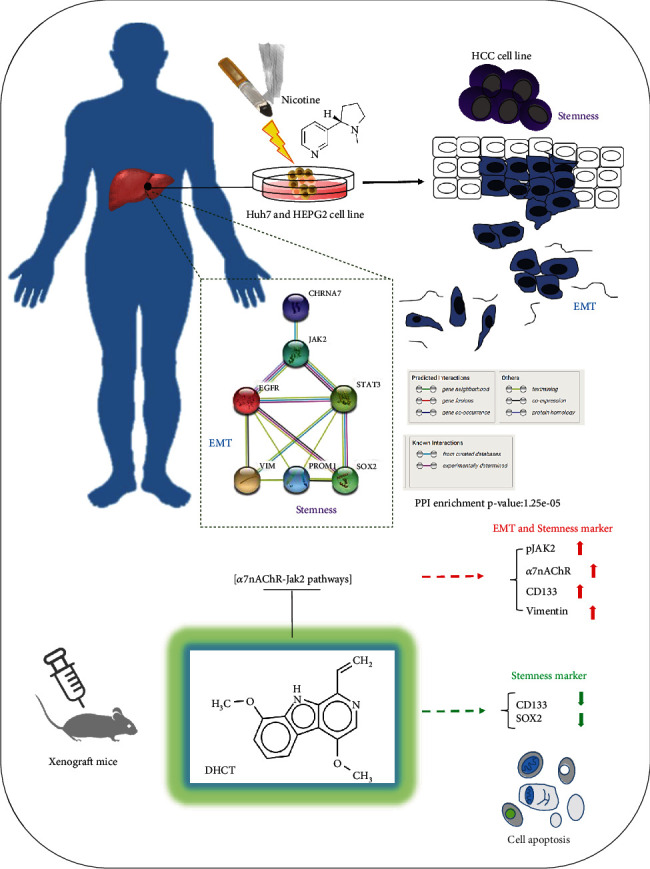
A pictorial abstract showing the natural compound dehydrocrenatidine regulating the a7nAChR-Jak2 signaling pathways attenuates nicotine-induced stemness and epithelial-mesenchymal transition in hepatocellular carcinoma and a series of experimental designs.

In conclusion, as shown in the pictorial abstract of Figure 7, it demonstrated that the natural compound dehydrocrenatidine attenuates nicotine-induced HCC cell stemness and EMT potential through a7nAChR-Jak2 signaling disruption and a series of experimental designs. Our results revealed that nicotine exposure-induced tumorigenicity in HCC cells by inducing the expression of *α*7nAChR and JAK2, which was effectively suppressed by DHCT treatment. This led to a reduction of CSC genes and antitumor efficacy in HCC cells. Thus, DHCT treatment could overcome/reverse the nicotine-induced progression of HCC cells, which may serve as a useful therapeutic strategy in patients with HCC.

## Data Availability

The datasets used and analyzed in the current study are publicly accessible as indicated in the manuscript.

## Additional Points

*Highlights*. (i) Natural compound dehydrocrenatidine attenuates nicotine-induced stemness in hepatocellular carcinoma. (ii) Natural compound dehydrocrenatidine attenuates nicotine-induced epithelial–mesenchymal transition in hepatocellular carcinoma. (iii) Natural compound dehydrocrenatidine regulating a7nAChR-Jak2 signaling pathways in hepatocellular carcinoma.

## Supplementary Materials

Supplementary Materials Supplementary Table S1: Western blot antibodies sheet. Supplementary Table S2: primer sequence in this study.
