# Supplementary material for: The Natural Compound Dehydrocrenatidine Attenuates Nicotine-Induced Stemness and Epithelial-Mesenchymal Transition in Hepatocellular Carcinoma by Regulating a7nAChR-Jak2 Signaling Pathways
Source: Dis Markers. 2022 Jan 24;2022:8316335. doi: 10.1155/2022/8316335 (PMC8803439; doi:10.1155/2022/8316335)
Supplement: Supplementary Materials — Supplementary Table S1: Western blot antibodies sheet. Supplementary Table S2: primer sequence in this study. [file 8316335.f1.docx]

**Supplementary Table S1. Western blot antibodies sheet.**

**
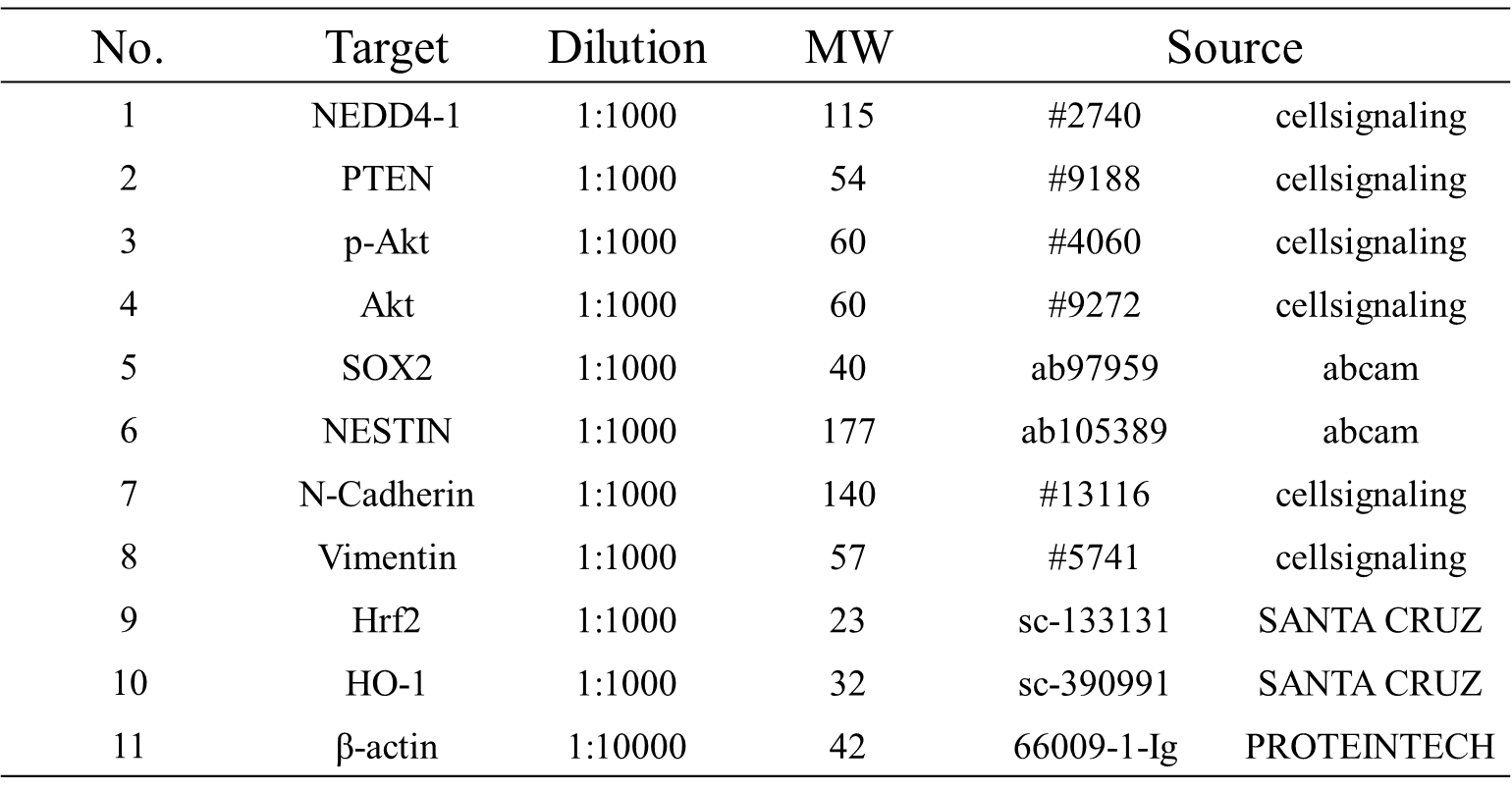
**

**Supplementary Table S2. Primer sequence in this study.**

| - Gene | - sequence | |
| --- | --- | --- |
| - Ki67 | - forward | - AGACAGCCACTCACCTCTTCAG |
|  | - reverse | - TTCTGCCAGTGCCTCTTTGCTG |
| - Cyclin D1 | - forward | - CGGTGAAACTCTGGCTAGACAG |
|  | - reverse | - GCAAACCGTAGATGCTCAGGGA |
| - JA | - forward | - GCAGCACTACTTCTTGACCACC |
|  | - reverse | - TCTGCTCCTGAGCATTGACGTC |
| - Alpha7-nAChR | - forward | - CTCTTCTGCCTGCTGCACTTTG |
|  | - reverse | - ATGGGCTACAGGCTTGTCACTC |
| - GAPDH | - forward | - ACGTTGTGTAGCTTATCAGACTG |
|  | - reverse | - AATGGTTGTTCTCCACACTCTC |
